# Efficacy of total transanal laparoscopic pull‑through and pure transanal endorectal pull‑through in the treatment of common‑type Hirschsprung disease

**DOI:** 10.20452/wiitm.2024.17914

**Published:** 2024-11-19

**Authors:** Guizhen Huang, Wenqian Huang, Chi Sun, Meng Li, Chaosheng He, Yi Su, Weili Xu, Suolin Li

**Affiliations:** Department of Pediatric Surgery, The First Affiliated Hospital of Xiamen University, School of Medicine, Xiamen University, Xiamen, China; Department of Pediatric Surgery, The Second Hospital of Hebei Medical University Shijiazhuang, Shijiazhuang, Hebei, China

**Keywords:** Hirschsprung disease, pure transanal endorectal pull-through, total transanal laparoscopic pull-through

## Abstract

**INTRODUCTION:**

Hirschsprung disease (HD) is a birth defect in which some of the intestinal nerve cells (ganglion cells) do not form completely. Improvements in laparoscopic skills among pediatric surgeons, along with technological advancements, have led to the widespread use of the natural orifice transluminal endoscopic surgery (NOTES) technique; however, reports on long-term outcomes and high-quality follow-up data on anorectal manometry in patients treated with this approach are scarce.

**AIM:**

We aimed to compare the short-term and long-term efficacy of 2 surgical approaches to the treatment of common-type HD: total transanal laparoscopic pull-through (TTLP, which falls under the category of NOTES), and pure transanal endorectal pull-through (PTEP; not classified as NOTES) in order to provide a reference for clinical strategy selection.

**MATERIALS AND METHODS:**

We retrospectively evaluated clinical data and follow-up results of 60 children with common-type HD who underwent TTLP or PTEP. The patients were divided into 2 equal-size groups according to the treatment method. Perioperative parameters were recorded, and regular follow-up was conducted by designated staff for over 10 years. The frequency and type of postoperative short- and long-term complications, pre- and postoperative anorectal manometry data, and daily bowel movement frequencies were recorded. The postoperative defecation function and quality of life scores were assessed and compared.

**RESULTS:**

The mean (SD) age of patients undergoing surgery was 16.75 (12.82) months in the TTLP group and 18.92 (11.55) months in the PTEP group. The incidence of perioperative and long-term complications did not differ between the groups. One month postsurgery, the TTLP group showed lower values of anorectal manometry indicators, as compared with the PTEP group. At 1 to 10 years postsurgery, except for lower anal resting pressure values in the TTLP patients, there was no significant difference in the anorectal manometry indicators between the groups. Both early and late postoperative defecation frequencies were similar between the 2 types of surgeries. Within the first 6 months postsurgery, the defecation function scores were higher in the TTLP group than in the PTEP group; however, after 1 year, there were no significant differences in these scores between the groups. The quality of life scores of the 2 groups showed no difference in the first 1 to 2 years of the surgery. However, the mean quality of life scores evaluated from 2 to 10 years postsurgery were higher in the TTLP group than in the PTEP group.

**CONCLUSIONS:**

TTLP for common-type HD not only contributes to early postoperative recovery, but also enhances the long-term quality of life, as compared with PTEP.

## INTRODUCTION

Hirschsprung disease (HD) is a congenital disorder caused by various factors that impede the migration of ganglion cells in the digestive tract during embryonic development. The absence of ganglion cells in the distal bowel leads to persistent spastic contractions. This results in functional intestinal obstruction, which requires surgical resection of the affected bowel segment for relief. Since the introduction of minimally invasive endosurgery in the 1980s, the application of laparoscopy in the surgical treatment of HD has become increasingly popular. Although initial reports^1^ did not gain immediate attention, widespread adoption followed after Georgeson et al^2^ demonstrated the feasibility of using laparoscopy for biopsy and mobilization before performing transanal pull-through procedures. Improvements in laparoscopic skills among pediatric surgeons and advancements in surgical devices have led to the development and growing application of a new technique, natural orifice transluminal endoscopic surgery (NOTES). However, reports on long-term outcomes and high-quality follow-up data on anorectal manometry in patients treated with this approach are scarce.^3^^; ^^4^ In the present study, we delineate and juxtapose 2 distinct surgical methodologies, total transanal laparoscopic pull-through (TTLP) and pure transanal endorectal pull-through (PTEP). The former surgery type falls under the category of NOTES, while PTEP surgery, although performed through natural body cavities, is not classified as NOTES.

## AIM

This retrospective analysis aimed to com‑ pare the early and late outcomes of TTLP and PTEP performed in children with common‑type HD to provide a basis for clinical decision‑making.

## MATERIALS AND METHODS

### Study population

The study included 60 children treated between 2009 and 2013 at the Second Hospital of Hebei Medical University and the First Affiliated Hospital of Xiamen University, People’s Republic of China. All participants presented typical clinical symptoms of HD, including delayed meconium passage and constipation. They were diagnosed with common-type HD, characterized by the absence of ganglion cells in the rectum and sigmoid colon. The children were randomly divided into the TTLP group and the PTEP groups, according to the treatment method, with 30 cases assigned to each group.

The inclusion criteria encompassed: 1) typical symptoms of megacolon; 2) diagnosis of a common type of megacolon based on lower gastrointestinal X-ray findings and biopsy of rectal mucosa; 3) age ranging from 6 months to 3 years; and 4) HD treated with the Soave procedure.

The following patients were excluded from the study: 1) individuals suffering from megacolon with short or long segments; 2) those lost to follow-up after surgery; 3) those aged below 6 months or over 3 years; 4) those treated with other surgical methods; and 5) individuals with comorbidities such as congenital heart disease.

### Case grouping method

First, we screened the patients based on age to ensure comparability. For selection of the surgical technique, we employed a rigorous randomization process to ensure the unbiased allocation of patients to receive TTLP or PTEP. The patients with odd hospital admission numbers were assigned to the TTLP group, while those with even hospital admission numbers were placed in the PTEP group. By the end of the study, both groups had more than 30 patients, providing a sufficient sample size to allow for detecting meaningful differences in treatment outcomes. To further enhance comparability between the groups, we employed a matching algorithm that considered the number of patients with available follow-up data and patient age. Subsequently, a lottery method was used to randomly select 30 patients from each group, ensuring an unbiased selection process. To minimize the risk of bias and maintain study integrity, the staff that gathered the follow-up data and anal manometry technicians were blinded to the surgical method employed in each case. This single-blind design ensured the elimination of any potential bias arising from the knowledge of the surgical technique used, further strengthening the validity of the study findings.

#### Surgical procedures

##### Total transanal laparoscopic pull-through

**Figure 1:**
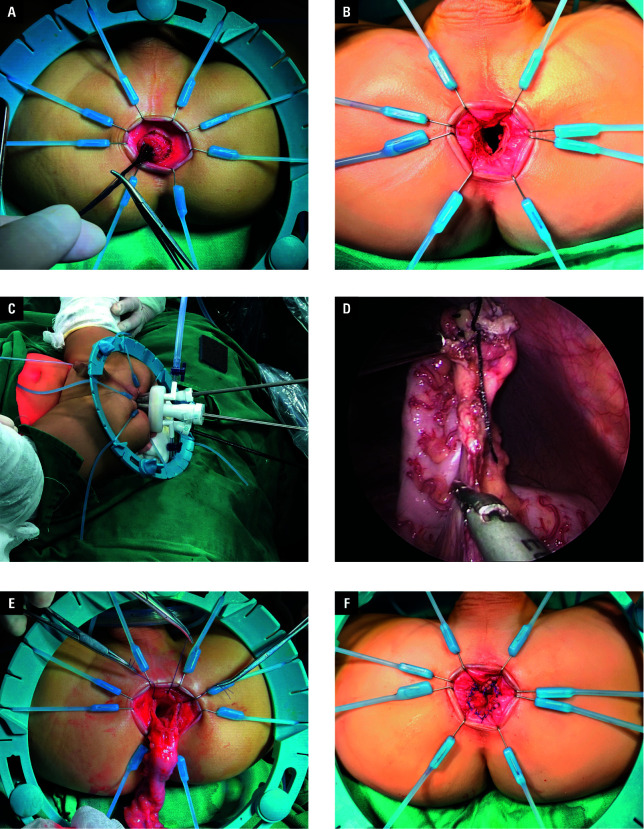
Steps of the total transanal laparoscopic pull-through procedure

In the TTLP group, combined general anesthesia with a caudal block was administered. The patient was placed in the supine position, and routine sterilization of the abdomen, perineum, and both legs was performed. The legs were wrapped in sterile drapes and placed apart. An anal speculum was used to dilate the anus. A precise oblique incision of 0.5 cm to 1 cm was made on the posterior rectal mucosal wall and on the anterior wall at a distance of 1.5 cm to 2 cm from the dentate line (Figure 1A). The rectal mucosal tube was dissected up to the peritoneal reflection, and the rectal muscle layer was transected to provide access to the pelvic cavity. The rectum was then severed at its upper margin, whereas the proximal stump was ligated and repositioned into the abdominal cavity. The distal rectum was then dissected and resected. A strategic “V” incision was made in the posterior wall of the rectal muscle sheath, preserving the anterior wall and both sides of the rectal muscle sheath below the peritoneal reflection (Figure 1B). A protective sleeve was threaded through the anal canal, extending between the pelvic floor and the anal aperture, and secured with a TriPort seal. Surgical instruments including a laparoscope, forceps, and an ultrasonic scalpel were introduced via the TriPort (Figure 1C). The sigmoid colon was repositioned using forceps, and the mesocolon was severed at its base with an ultrasonic scalpel, along with the peritoneum adjacent to the descending colon and the splenic flexure. Consequently, the colon was freed for a length of 10 cm to 15 cm above the transition zone (Figure 1D). The mesenteric blood vessels were carefully aligned, and the liberated colon was drawn through the anus, trimmed, and submitted for histological examination. After ensuring that the mesentery was untwisted, the pulled-down colon was fixed to the rectal muscle sheath with quadruple sutures (Figure 1E). The edge of the descended colon was sutured to the remaining rectal mucosa using a bioabsorbable suture (Figure 1F). A mushroom catheter covered in paraffin jelly was inserted above the anastomosis to compress the pelvic floor and facilitate drainage.

**A** – peeling of the rectal mucosa; **B** – “V” cut in the posterior wall of the rectal muscle sheath; **C** – placement of a TriPort designed for children through the anus to establish a transanal laparoscopic operation channel; **D** – severing of the secondary blood vessels supplying the sigmoid colon using an ultrasound knife; **E** – discontinuous fixing of the pulled colonic and rectal muscle sheaths, anastomosis of the dissected edge of the colon with the dissected edge of the rectal mucosa; **F** – anastomosis of the rectum and colon

##### Pure transanal endorectal pull-through

**Figure 2:**
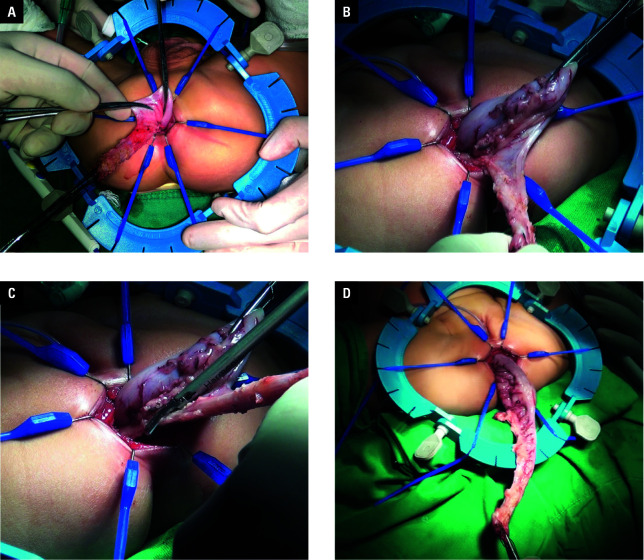
Steps of pure transanal endorectal pull-through procedure

For the PTEP approach, the administration of anesthesia, patient positioning, and steps of the transanal endorectal Soave procedure were analogous to those described above for the TTLP surgery. Following the dissection of the rectal muscle sheath above the peritoneal reflection, the rectum was meticulously pulled downward through the anorectal muscle sheath. This process involved careful stripping of the rectum and mesocolon off the sigmoid colon, ligating, and severing the marginal blood vessels until the freed colon extended 10 cm to 15 cm above the transition zone of the normal colon segment. Subsequently, the liberated colon was excised and subjected to external examination. The distal end of the normal colon was then precisely sutured to the preserved mucosal fringe just above the dentate line, ensuring the anal tube remained in place (Figure 2).

**A** – exfoliation of the rectal mucosa and severing of the muscular sheath of the rectum; **B** – pulling out of the colon after severing of the rectal sheath; **C** – severing of the mesentery at the intestinal tube margin; **D** – liberating the colon to the dilated segment, pulling the colon out through the anus, removal of the diseased bowel, and anastomosis of the proximal intestinal cut with the rectal cut margin

### Data collection

Regular outpatient follow-up assessments were conducted by designated staff for over 10 years. Complications that occurred within the first 2 years were classified as short-term outcomes, while those identified after 2 years were considered mid- to long-term outcomes. Both pre- and postoperative anorectal manometry data and the average daily frequency of bowel movements were recorded at various time points.

### Enterocolitis diagnosis

Enterocolitis was diagnosed when a child presented abdominal pain, diarrhea, bloating, and fever. The laboratory findings corroborating the diagnosis included leukocytosis on complete blood count or fecal leukocytes on stool analysis.

### Evaluation methods

Bowel function was assessed using the method proposed by Reding et al,^5^ considering a number of criteria. The first criterion was bowel evacuation, where 1 point was awarded for at least 1 bowel movement per day, 0.5 point for at least 3 movements per week, and 0 points for fewer than 3 movements per week. The second evaluated factor was abdominal distension, where 1 point corresponded to no distension, 0.5 point to occasional distension, and 0 to daily or persistent distension. Another assessed element was soiling, with 2 points allocated if it occurred rarely or never, 1 point if it occurred fewer than 3 times per week, and 0 points if it occurred 3 or more times per week. Subsequently, fecal incontinence was evaluated, where 1 point meant it never occurred and 0 points indicated that it occurred occasionally. The maximum number of points to score was 5, which was considered excellent, 2 to 3.5 points corresponded to moderate, and a score of 0 to 1.5 points was classified as poor bowel function. Quality of life was evaluated postoperatively using a validated pediatric-specific instrument designed for the assessment of postanorectal surgery outcomes^6^ (Table 1).

**Table 1:** Tool for quality of life assessment after anorectal surgery in children

Scoring item	Clinical feature	Score, points^a^
Fecal incontinence and soiling	Total incontinence	0
	Frequent incontinence, fecal soiling during diarrhea	1
	Frequent fecal soiling	2
	Occasional fecal soiling	3
	None	4
School attendance	Inability to attend school or frequent absence	0
	Occasional absence	1
	Normal	2
Diet	Restricted	0
	Occasional restrictions	1
	Unrestricted	2
Social interactions	Poor	0
	Occasional failure	1
	Normal	2
Occurrence of bowel problems	Frequently	0
	Occasionally	1
	Never	2

**a **Poor: 0–4 points; moderate: 5–8 points; excellent: 9–12 points

### Statistical analysis

Data analysis was performed using SPSS software, version 27.0 (IBM Corp., Armonk, New York, United States). Quantitative data that followed a normal distribution were expressed as mean (SD), and comparisons between the groups were performed with the *t* tests. Categorical data were presented as numbers and percentages, and differences between the groups were assessed using the Fisher exact test. A *P* value of less than 0.05 was considered to denote statistical significance.

### Ethics statement

The study was conducted in accordance with the principles set out in the Declaration of Helsinki, and the study protocol was approved by the ethics committees of Hebei Medical University and Xiamen University (2013046).

## RESULTS

### Clinical data

The study included 46 boys and 14 girls, aged from 6 to 36 months. The cohort was divided into 2 groups, according to the treatment method. Both groups were comparable in terms of sex distribution, age, and weight. The mean (SD) age of the TTLP patients was 16.75 (12.82) months, and of the PTEP patients, 18.92 (11.55) months (Table 2).

**Table 2:** Clinical characteristics of the study population

Parameter		TTLP (n = 30)	PTEP (n = 30)	*P* value
Sex, n (%)	Boys	22 (73.3)	24 (80)	0.54
	Girls	8 (26.7)	6 (20)	
Age, mo, mean (SD)		16.75 (12.82)	18.92 (11.55)	0.94
Weight, kg, mean (SD)		12.38 (3.58)	13.47 (3.29)	0.22

Abbreviations: TTLP, transanal laparoscopic pull-through; PTEP, pure transanal endorectal pull-through

### Short-term complications

In the TTLP group, 3 patients (10%) developed short-term complications. One patient experienced rectal muscle sheath abscess coupled with partial dehiscence of the suture site, which resolved following conservative management. Additionally, 2 cases of postoperative enterocolitis were recorded, both of which were successfully managed through anal dilation and bowel training. In the PTEP group, 8 patients (26.7%) experienced short-term complications. These included 3 cases of enterocolitis, 1 case of fecal incontinence, and 1 case of recurrent constipation due to incomplete resection of the aganglionic segment, necessitating a laparoscopic-assisted colonic pull-through procedure for resolution. Furthermore, 2 patients exhibited anastomotic dehiscence that required reoperation with subsequent stoma formation, and 1 presented a 270-degree twisting of the pulled-through bowel leading to partial anastomotic dehiscence and incomplete obstruction, which required corrective laparoscopic surgery. The incidence of short-term complications did not differ between the 2 groups (Table 3).

**Table 3:** Comparison of short- and long-term postoperative complications

Complications	TTLP (n = 30)	PTEP (n = 30)	*P* value
Short-term			
Total	3 (10)	8 (26.7)	0.095
Anastomotic rupture	1 (3.3)	2 (6.7)	
Enterocolitis	2 (6.7)	3 (10)	
Recurrent constipation	0	1 (3.3)	
Fecal incontinence	0	1 (3.3)	
Intestinal obstruction due to lower colonic torsion	0	1 (3.3)	
Long-term			
Total	1 (3.33)	4 (13.33)	0.16
Sporadic enterocolitis	1 (3.33)	0	
Fecal soiling	0	3 (10)	
Recurrent constipation due to anal stenosis	0	1 (3.33)	
Patients requiring reoperation			
Total	0	5 (16.67)	-
Short-term	0	4 (13.33)	
Long-term	0	1 (3.33)	

Data are presented as number (percentage) of patients.

Abbreviations: see Table 2

### Mid- to long-term complications

In the TTLP group, 1 patient was reported to exhibit occasional colitis at the 2-year follow-up. No long-term complications, such as fecal incontinence or anal stricture, were observed in this group, resulting in a complication rate of 3.33%. In the PTEP group, 3 patients continued to experience persistent soiling due to damage at the dentate line of the posterior wall (including 1 case identified as malformed fecal incontinence). Additionally, 1 patient had unsatisfactory outcomes in the form of anal dilation and intractable constipation necessitating further surgical intervention. Consequently, the long-term complication rate in the PTEP group reached 13.33%. The difference in the prevalence of long-term complications between the 2 groups was not significant (Table 3).

### Bowel movement frequency and bowel function

No significant differences between the groups were observed in terms of the average number of daily bowel movements during both the perioperative and long-term periods. However, within 6 months of the surgery, the TTLP group exhibited a higher bowel function score than the PTEP group. Intergroup differences in the bowel function scores evaluated at 1-year follow-up and later were no longer significant (Table 4 ).

**Table 4:** Quality of life scores, bowel function score, and anorectal manometry results at different follow-up time points

Time point	PTEP	TTLP	*P *value
Bowel movements, n			
1 month	8.61 (2.87)	8.71 (1.99)	0.85
3 months	5.32 (2.44)	4.69 (2.9)	0.37
6 months	3.63 (2.26)	3.79 (1.96)	0.77
1 year	3.42 (2.15)	3.24 (1.48)	0.71
5 years	2 (1.11)	2.24 (1.14)	0.41
10 years	1.91 (0.83)	1.71 (0.91)	0.38
Bowel function score^a^, points			
1 month	2.18 (0.61)	2.67 (0.57)	0.002
3 months	3.21 (0.82)	3.77 (0.7)	0.006
6 months	4.06 (0.79)	4.58 (0.92)	0.022
1 year	4.04 (0.54)	4.2 (0.16)	0.12
5 years	4.49 (0.68)	4.64 (0.52)	0.34
10 years	4.64 (0.26)	4.75 (0.46)	0.26
Quality of life score, points			
2 years	12 (0.25)	11.64 (1.15)	0.1
10 years	10.06 (2.11)	11.33 (0.8)	0.002
Anal high-pressure zone length, mm			
Preoperative	25.1 (4.07)	23.97 (2.99)	0.23
1 month	22.26 (3.28)	19.17 (1.95)	<0.001
6 months	20.15 (2.35)	17.19 (2.16)	<0.001
1 year	16.69 (3.22)	17.28 (1.2)	0.35
5 years	18.82 (4.53)	19.33 (2.41)	0.59
10 years	20.21 (3.67)	21.09 (1.96)	0.25
Anal resting pressure, kPa			
Preoperative	22.52 (7.84)	20.86 (8.28)	0.43
1 month	14.27 (0.93)	11.96 (1.11)	<0.001
6 months	11.68 (1.98)	9.2 (3.77)	0.02
1 year	10.81 (7.24)	7.36 (4.81)	0.03
5 years	9 (4.02)	7.46 (2.8)	0.09
10 years	4.07 (1.02)	4.03 (1.33)	0.94
Rectal resting pressure, kPa			
Preoperative	3.38 (1.25)	3.46 (0.98)	0.78
1 month	2.46 (1.03)	2.01 (0.52)	0.04
6 months	1.72 (0.5)	1.74 (0.44)	0.87
1 year	1.14 (2.77)	2.14 (1.06)	0.07
5 years	0.98 (1.35)	1.45 (2.46)	0.36
10 years	0.1 (0.26)	0.06 (0.73)	0.78

Data are presented as mean (SD).

**a **Calculated using the method proposed by Reding et al^5^

Abbreviations: see Table 2

### Quality of life scores

During the first 2 years of follow-up, there were no significant differences in the quality of life scores between the 2 groups. However, within the 2 to 10 years postoperative interval, the mean (SD) quality of life score for the TTLP group was 11.33 (0.8) points, while in the PTEP group it was 10.06 (2.11) points (*P* = 0.002) (Table 4 ).

### Anorectal manometry results

Before the surgery, there were no significant differences between the 2 groups in terms of preoperative anal resting pressure, rectal resting pressure, and the length of the high-pressure zone in the anal canal. One month after the surgery, the TTLP group showed lower values of these 3 parameters, as compared with the PTEP group. At the 6-month follow-up, the PTEP group still exhibited significantly higher anal resting pressure and greater high-pressure zone length than the TTLP group, while the difference in rectal resting pressure was no longer significant. From 6 months to 1 year postsurgery, there were no significant differences in the evaluated anorectal manometry indicators, except for the anal resting pressure. There were no differences in long-term anorectal manometry results between the groups (Table 4 ).

## DISCUSSION

The surgical approach for treating HD has evolved significantly over the years. In 1998, De la Torre et al^7^ discovered that the rectosigmoid colon could be mobilized and pulled out through the anus alone during laparoscopic-assisted surgery, which gained immediate popularity. The advantages of PTEP include avoiding laparotomy, minimizing surgical trauma, and simplifying the procedure, especially through the transanal route, creating a new pathway that exclusively isolates the rectum and sigmoid colon from the perineal direction. Although PTEP is minimally invasive and cosmetically appealing, it presents certain challenges. In the cases of an indistinct transition zone, PTEP necessitates liberation of the bowel segment prior to biopsy, and the subsequent wait for frozen section results extends surgery duration. Additionally, the perineal-only mobilization of the rectum and sigmoid colon complicates the assessment of pathological extents and demands meticulous management of the mesenteric margin vessels—a task that is both time-consuming and complex (mean [SD] duration, 131.62 [24.92] min vs 106.35 [17.46] min, respectively, for PTEP and TTLP; *P* <⁠0.01). Forceful anal retraction may also cause damage to the external sphincter muscle. The PTEP approach is limited to releasing the rectum and sigmoid colon without ensuring full mobilization of the descending colon. Tension at the anastomosis can lead to a loss of the anorectal angle and even result in anastomotic dehiscence or severe complications and infections. In patients with long-segment HD, while the Soave procedure can be performed with laparoscopic assistance, it is difficult to achieve complete resection of all affected intestinal segments solely through PTEP. Furthermore, this technique frequently results in surgical failure due to excessive tension at the anastomosis and subsequent postoperative intestinal retraction. Therefore, opting for the PTEP method in the context of long-segment HD is deemed inappropriate. In this study, 3 patients (10%) experienced such complications as intestinal retraction, a notably high incidence rate. Additionally, due to narrow infant anorectal diameter and thickened and dilated bowel above the transitional zone in young children, excessive force during mesenteric handling through the purely transanal approach can overstretch and damage the sphincter. Inadvertent repeated maneuvers may cause trauma to the dentate line and strip the residual rectal mucosa on the posterior wall. This can lead to long-term postoperative fecal soiling, and even incontinence when stools are not formed, as evidenced by a high incidence of partial incontinence in the PTEP group, reaching 10%. However, we observed no significant difference in the incidence of short- and long-term complications between the 2 groups. This finding is consistent with the results of a recently published meta-analysis.^8^

With the advancement of endoscopic minimally invasive techniques, NOTES has become a focus of current research. In 2009, Velhote et al^9^ reported the first successful treatment of HD using transanal laparoscopic-assisted colon extraction, which was encouraging for clinicians. Building upon the foundations of conventional laparoscopic-assisted and single-incision transumbilical laparoscopic-assisted HD surgery, the authors successfully performed 30 curative surgeries in patients with common-type HD entirely via the transanal natural orifice under laparoscopic assistance.

Laparoscopic assistance allows for comprehensive visualization of intra-abdominal conditions, enabling accurate determination of the disease extent and severity. A frozen section biopsy can be performed beforehand for definitive excision of the affected bowel, thanks to which freeing the bowel mesentery from the secondary vascular arch is simplified and accelerated, significantly reducing operative time and blood loss during surgery. This technique enables full mobilization of the pulled-down bowel with well-preserved blood supply and completing the rectocolonic anastomosis without twists or tension, thereby reducing postoperative complications and noticeably shortening postoperative hospital stays.

The use of a TriPort protective sheath minimizes trauma to the dentate line, residual rectal mucosa, and muscular cuff during the procedure, maximally preserving defecation sensation. While most patients undergoing a pull-through procedure for HD typically do not develop long-term complications, a small number experience persistent bowel problems, such as constipation or recurrent enterocolitis.^10^

In our study, 4 patients in the PTEP group experienced bowel problems in the long term, with 1 case requiring a subsequent operative procedure. However, no long-term complications were noted in the TTLP group. Furthermore, life quality scores measured at the 10-year follow-up were superior in the TTLP group, as compared with the PTEP group. This indicates that adequate bowel mobilization and protection of the dentate line not only facilitate early postoperative recovery, but also enhance long-term quality of life.

Anorectal manometry has been in use for over a century, and holds high specificity in the diagnosis of HD, making it the primary screening method in pediatric surgery. In this paper, we evaluated and compared 3 critical indicators: resting rectal pressure, resting anal pressure, and the length of the high-pressure zone in the anal canal. During the perioperative period, these 3 indicators in both groups remained relatively high, but significantly lower than the presurgery values. In the short-term postoperative period, the PTEP group showed significantly higher resting anal pressure and anal canal high-pressure zone length than the TTLP group, while no significant difference was observed in the resting rectal pressure. In the medium- to long-term follow-up, the manometric results of the rectum showed no differences between the 2 groups. This indicates that the short-term recovery of rectal anal pressure in the TTLP patients was notably better than that recorded in the PTEP group, while there was no significant difference in the long-term recovery between the 2 surgical approaches. The temporary discrepancies in rectal anal manometry may be due to a certain degree of sphincter damage in the PTEP group, which does not heal rapidly, but gradually improves with anal dilation and defecation training.

Onishi et al^11^ suggested that PTEP should be performed before the patient reaches 6 months of age to ensure achievement of optimal bowel function. The surgical technique of this procedure must be refined based on a detailed understanding of the anatomy.^12^ The present study also comprised medium- to long-term clinical evaluations of bowel control in both groups, showing that postoperative bowel function recovery in the TTLP group was superior to that observed in the PTEP group. This confirms the effectiveness of the TTLP protective sheath in preserving the dentate line and distal rectal mucosa, which maximally preserves the internal sphincter within the rectal muscular cuff, thereby enabling better control of voluntary defecation. Furthermore, with laparoscopic assistance, the involved bowel can be better visualized and fully freed down the descending colon. This ensures the absence of tension in the pulled-down bowel, effectively reducing the resting pressure of the anal canal and the length of the anal high-pressure zone, thereby improving bowel function recovery.

Quality of life scoring is a subjective assessment completed by patients themselves, reflecting their personal perceptions of their diet-related issues, bowel function, ability to attend school, and social interactions. At 2 years postoperatively, there was no difference in the scores between the 2 groups. However, 10 years after the surgery, the TTLP group recorded higher scores. This discrepancy is likely caused by the fact that the 2-year scores were assigned by patients’ family members due to their young age, while the scores provided 2 to 10 years postsurgery were self-reported by the patients. Thus, we postulate that the long-term scoring results more accurately reflect the patients’ experiences and overall quality of life.

The higher scores of the TTLP group may be attributed to the fact that transanal laparoscopic surgery, assisted by a TriPort protective sleeve, can perfectly preserve the anterior rectal wall mucosa and the dentate line. This preservation maximizes the defecation reflex function and allows for better control of bowel movements, enhancing overall quality of life.

## CONCLUSIONS

The TTLP procedure demonstrates significant advantages over the PTEP approach in the treatment of common-type HD. These benefits are reflected in both short- and long-term outcomes, such as quicker postoperative recovery and improvement of the overall quality of life.
